# Twitter and Public Health (Part 2): Qualitative Analysis of How Individual Health Professionals Outside Organizations Use Microblogging to Promote and Disseminate Health-Related Information

**DOI:** 10.2196/publichealth.6796

**Published:** 2017-10-04

**Authors:** Mark Hart, Nichole Stetten, Sabrina Islam, Katherine Pizarro

**Affiliations:** ^1^ Department of Behavioral Science and Community Health University of Florida Gainesville, FL United States

**Keywords:** Twitter, social media, public health, technology transfer, diffusion of innovation

## Abstract

**Background:**

Twitter is the most popular form of microblogging that is being utilized in public health to engage audiences and to communicate health-related information. Although there is some research showing the various forms of Twitter use in public health, little is known about how individual public health professionals are using their personal Twitter accounts to disseminate health information.

**Objective:**

The purpose of this research was to categorize public health professionals’ tweets to evaluate how individual public health professionals are furthering the mission of public health.

**Methods:**

Twitter accounts held by public health professionals were identified, and researchers proceeded to record 6 months’ worth of each individual’s Twitter feed. During the 6-month period, a total of 15,236 tweets were collected and analyzed using the constant comparison method.

**Results:**

A total of 23 tweet categories among the 15,236 tweets were initially identified. Some of the most common topics among the 23 categories included the following: health nutrition (n=2008), conferences (n=815), Ebola (n=789), Affordable Care Act (ACA)/health care (n=627), and social justice (n=626). Each of these categories were then stratified into one of four themes: (1) informing and educating, (2) monitoring health statuses and trends, (3) social justice, and (4) professional development.

**Conclusions:**

Using Twitter, public health professionals are helping dispel misinformation through education and by translating technical research into lay terms, advocating for health inequalities, and using it as a means to promote professional development.

## Introduction

In a recent paper published in JPHS we described how public health professionals use Twitter for professional development [1]. In the current paper, we describe how public health professionals disseminate health related information using Twitter. Facebook, Twitter, Instagram, Yik Yak, Snapchat, and YouTube are just a few of the social networking sites (SNSs) that 65% of adults use daily in the United States [2,3]. Each platform offers a unique way to disseminate information, share opinions, and connect with others around the world in a matter of seconds [2]. Microblogging, a subsection of SNSs, is defined as “short, frequent posts” or electronic word of mouth [4-6]. Through microblogging, individuals not only share personal mementos, opinions, political information, and news but also promote products and information, thereby raising awareness for causes or charities [4-6]. Research shows that microblogging is powerful in convincing/rallying other individuals because of immediacy, its far reach to individuals around the world, and is seen as credible because it appears in a print format [6]. As a result of its strong influence, many companies and organizations have adopted microblogging to disseminate information about their company or organization and to promote events and products [4-6]. With such a strong influence and reach, it is important to look at how public health organizations and professionals are using this influence to potentially spread credible knowledge and information to the public as well as a means of professional development.

The most popular form of microblogging, with 313 million active users, occurs through Twitter [7,8]. Twitter allows users to post tweets up to 140 characters or less, as well as links, pictures, and videos after the tweet [7]. The majority of Twitter accounts are public, so any individual can access information on another account simply by following that account [7]. Twitter users are mostly individuals in the age group of 18 to 29 years, who are statistically African American or Hispanic, live in urban areas, and have a higher participation rate than most other SNSs [7]. These users are a challenging population to reach in public health, making Twitter an ideal resource for public health organizations and individuals to focus their efforts [7]. Public health organizations currently use Twitter for (1) informing and education, (2) monitoring health status and trends, (3) surveillance and information in disasters, and (4) professional development [7,9].

Public health organizations disseminate information and education by tweeting about various health-related topics [7,10]. Local health departments often tweet information about tobacco cessation resources, events, frequently asked questions about immunizations, and other popular health-related topics. Twitter has also been used as a means to provide sex education and to promote the use of condoms by tweeting facts on sexual health and information about local clinics that provide free condoms [10]. When monitoring health statuses and health trends, also called syndromic surveillance, health departments can search for tweets using keywords or hashtags such as “sick,” “flu,” “dental pain,” and “food poisoning.” This allows health departments to identify the geographical area from where these tweets are being posted and to map instances of a potential flu outbreak, food contamination, or an area in need of dental services in real time [7,11,12]. Furthermore, health organizations use surveillance and information during disasters by tweeting updates about current local crises such as flooding, fires, hurricanes, and tornadoes [7]. Besides sending information, health organizations can also collect information by searching for geotagged tweets that indicate where emergency relief should focus their attention [7].

Health organizations are also beginning to use Twitter as a source of professional development by tweeting updates while at a conference or an important meeting, thereby allowing other organizations and health professionals to receive updates on the current work and research being conducted [7]. Professional development is also being facilitated via the creation of journal clubs by certain organizations, a Web-based format on Twitter that allows health professionals to tweet questions and responses to the paper as well as the author of the paper to respond to other health professionals in real time [13]. Although public health organizations are adapting to the current social media trends, the current research being conducted focuses primarily on organizations and excludes how individual public health professionals are disseminating health information. This study aims to target these public health professionals’ tweets over a 6-month period to evaluate how individuals are furthering the mission of public health.

## Methods

When examining the implementation of new technology, Roger’s diffusion of innovation model is commonly used. Although Twitter was founded in 2006 and has gone through the full diffusion of innovation cycle (innovators, early adopters, early majority, late majority, and laggards), the diffusion of innovation model can be used to examine how new ideas and uses of Twitter spread throughout the population or a subpopulation/group. For example, public health organizations did not start using Twitter until more recently because of network filter blocks, but they created new uses for Twitter such as syndromic surveillance [7,11,12]. Public health organizations (eg, Centers for Disease Control and Prevention [CDC], local health departments, and National Institutes of Health [NIH]) are farther along the diffusion of innovation cycle, whereas individual public health professionals are just beginning to use Twitter for more than personal usage. These public health professionals can be considered early adopters, as they are expanding beyond the scope of personal usage and using their credentials to identify themselves as an authority and the field and to disseminate public health–related information outside a specific organization. Examining these tweets allows for the identification of information that public health professionals hope to disseminate to colleagues and eventually, to the general population.

### Data Collection

Participants were chosen through Twitter’s search function using the terms “public health practitioner,” “MPH” (master’s in public health), “public health,” and “APHA” (American Public Health Association). After individuals were identified as public health professionals, participants were chosen based on a set of inclusion and exclusion criteria. Inclusion criteria were as follows: the individual was a public health professional and had to have a minimum of 300 followers. Exclusion criteria were as follows: the individual could not be a part of an academic institution, and it could not be an organization’s Twitter page (eg, CDC, local health departments, and NIH). Overall, 220 public health professionals were chosen to examine their tweets during a 6-month period from October 1, 2014 to March 31, 2015. A total of 15,236 tweets were collected and then analyzed using the constant comparison method.

### Data Analysis

The constant comparison method was used to analyze the tweets to reduce the data into manageable units and coded information [14-16]. The process began with open coding, which can be defined as “the process of breaking down, examining, comparing, conceptualizing, and categorizing data,” where 2 trained researchers (NS and MH) open-coded all the tweets and discovered major themes [14-16]. The tweets were then selectively coded into those major themes by the same 2 trained researchers (MH and NS; [14-16]). Open coding was done by hand versus using keyword searches through data mining software to take on the full context of the tweets/posts.

## Results

The constant comparison method initially revealed 23 different tweet categories among the 15,236 tweets analyzed, as displayed in Table 1. Each of these categories were then analyzed and coded into four separate themes: informing and education, monitoring health status and trends, social justice, and professional development (Textbox 1).

**Table 1:** Tweet categories of public health professionals.

Tweet category	Tweets, n
Non-public health–related	4032
Health nutrition	2008
Other	1885
Conference/Forum/APHA^a^	815
Ebola	789
Noninfectious diseases	728
ACA^b^/Health care	627
Violence/Safety/Social justice	626
Health law and policy	567
Technology/Innovation	553
Environmental health/Factors	380
Charity/Organizations/NPO^c^	346
Vaccines	250
Education and literacy	233
Global famine/Water	196
Emergency/Emergency preparedness	176
Global poverty/Homelessness	170
Infectious diseases	158
Mental health	156
HIV^d^/AIDS^e^	143
Smoking/Tobacco/Marijuana	143
Medications/Drugs and alcohol	143
Influenza	112
Total	15,236

^a^APHA: American Public Health Association.

^b^ACA: Affordable Care Act.

^c^NPO: nonprofit organization.

^d^HIV: human immunodeficiency virus.

^e^AIDS: acquired immunodeficiency syndrome.

**Tweet themes of public health professionals and underlying categories.**

Tweet theme: Informing/Education

Categories

- Ebola
- Human immunodeficiency virus (HIV)/acquired immunodeficiency syndrome (AIDS)
- Affordable Care Act (ACA)/Health care
- Health law and policy
- Emergency/Emergency preparedness
- Environmental health factors
- Health and nutrition

Tweet theme: Monitoring health status/Trends

Categories

- HIV/AIDS
- Health and nutrition
- Influenza
- Smoking/Tobacco/Marijuana
- Technology/Innovation
- Infectious diseases
- Noninfectious diseases

Tweet theme: Social justice

Categories

- Global poverty/Homelessness
- Global famine/Water
- Charity/Organizations/American Public Health Association (APHA)
- Education and literacy
- Mental health
- Violence/Safety/Social
- Non-public health–related

Tweet theme: Professional development

Categories

Conference/Forum/APHA

### Informing and Education

**Figure 1:**
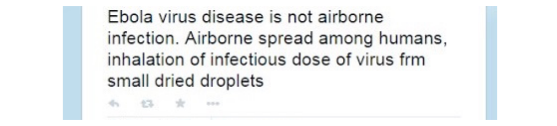
Public health professional dispels panic and myths during Ebola panic.

Informing and education tweets centered around informing and educating the public on various aspects and updates in public health. Within the theme, there were seven major topic areas covered: Ebola; human immunodeficiency virus (HIV)/acquired immunodeficiency syndrome (AIDS); Affordable Care Act (ACA)/health care; health law and policy; emergency/emergency preparedness; environmental health factors; and health and nutrition (Textbox 1). During the 6-month period of tweets collected, panic about Ebola was prevalent, and many public health professionals tweeted information to dispel panic and myths (see Figure 1).

**Figure 2:**
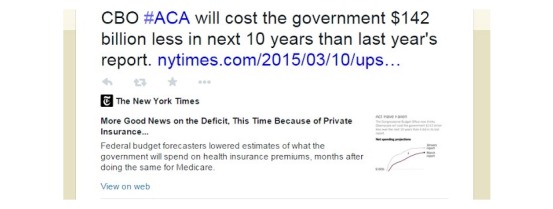
Public health professional dispels misinformation about the Affordable Care Act.

Another area where public health professionals tweeted information to dispel misinformation was about the ACA (see Figure 2).

Although social media has drastically changed the way of communication, it has also created a way to spread misinformation quickly. As public health professionals have the credentials/authority tied with their Twitter accounts, they can dispel misinformation as well as spread other important health information to the public at a rapid rate.

**Figure 3:**
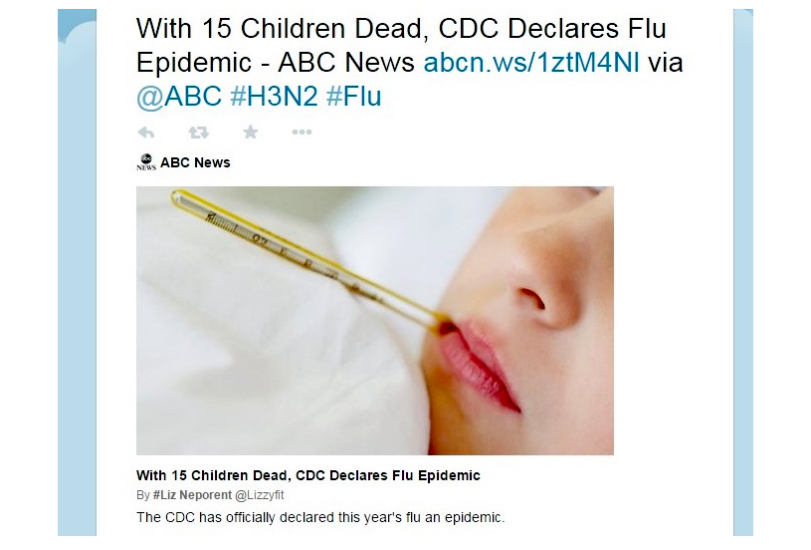
Public health professional disseminates information about flu epidemic.

**Figure 4:**
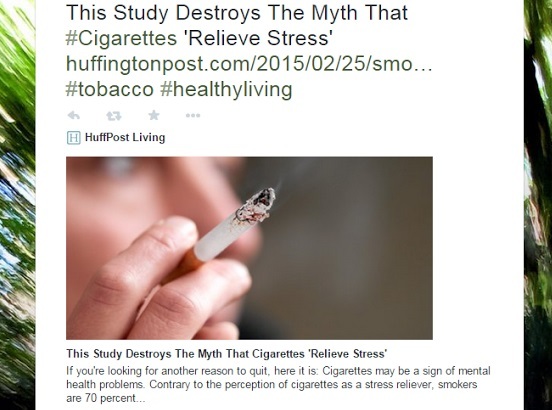
Public health professional shares a study about cigarettes with followers.

### Monitoring Health Status and Trends

Tweets identified as monitoring health status and trends were not using surveillance techniques, instead they were informing the public of updates on specific health statuses and trends (see Figures 3 and 4). Within the theme, there were seven major topic areas covered: HIV/AIDS; health and nutrition; influenza; smoking/tobacco/marijuana; technology/innovation; infectious diseases; and noninfectious diseases (Textbox 1).

Through the word limit function in Twitter, public health professionals are forced to condense important information into 140 characters, making these important updates on specific health statuses and trends more relatable to the lay population.

### Social Justice

**Figure 5:**
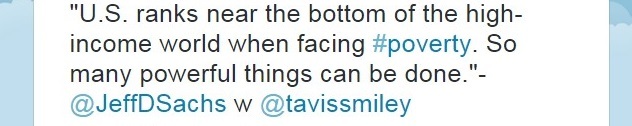
Public health professional speaks about US poverty.

**Figure 6:**
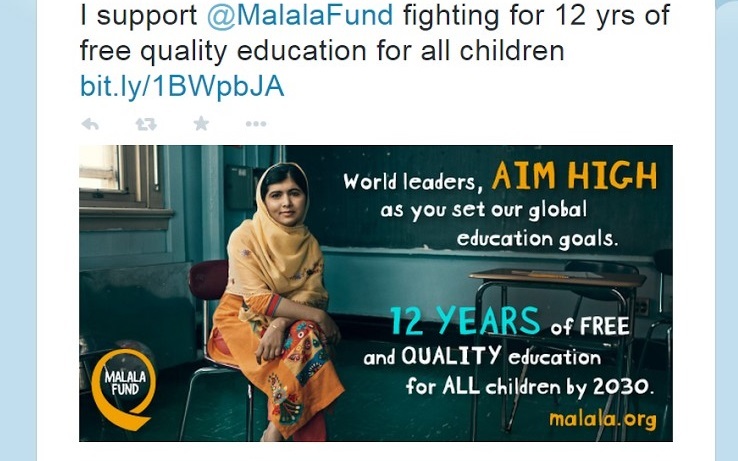
Public health professional shows support for an organization.

Social justice tweets focused on raising awareness and support for various public health issues (see Figures 5 and 6). Within the theme, there were seven major topic areas covered: global poverty/homelessness; global famine/water; charity/organizations/APHA; education and literacy; mental health; violence/safety/social justice; and non-public health–related (Textbox 1).

Twitter provides a space for public health professionals to share items they are passionate about, within or outside, their field. This platform also allows them to connect and collaborate with other professionals who are interested in the same social justice issues.

### Professional Development

**Figure 7:**
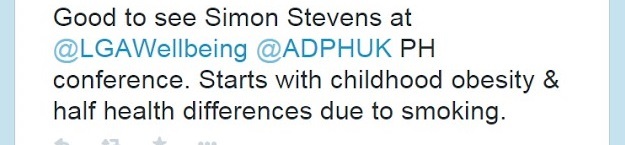
Public health professional shares his/herconference experience.

**Figure 8:**
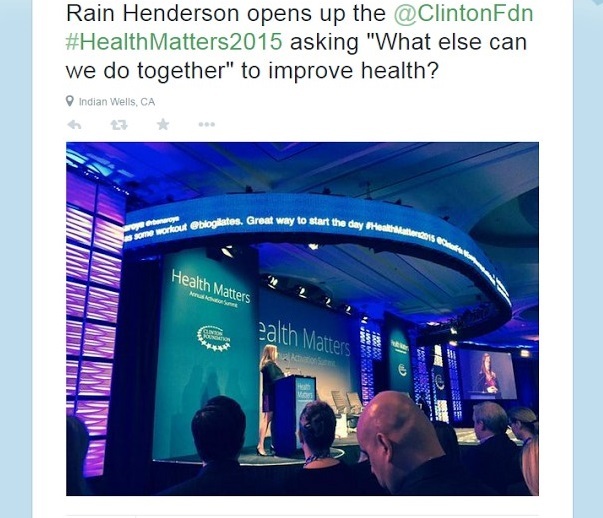
Public health professional shares a photo of a conference.

Professional development occurred when health professionals tweeted new public health information obtained at conferences and forums, as well as with the exchange of sources of scientific literature back and forth between other public health professionals and the general public (see Figures 7 and 8). This particular theme contained only one tweet category: conference/forum/APHA (Textbox 1).

Twitter provides a unique platform for professional development as budget cuts decrease the opportunity to connect and collaborate with other public health professionals.

## Discussion

### Principal Findings

Similar to public health organizations, public health professionals are also using Twitter to inform and educate the public. These results are not surprising, as one of the main tenets of public health is to “educate and inform” [7]. This information and education on social media is crucial, as Twitter’s main users (aged 18 to 29 years), do not obtain “news” from regular media (newspapers, magazines, and television) but from social media [17]. As social media has become a main resource of knowledge for many, so has misinformation. With the credibility seen in microblogging, it is important for public health professionals with credentials/authority to dispel this misinformation among the public [6]. The results show public health professionals attempting to combat misinformation on Ebola.

Public health organizations use Twitter to monitor health status and trends through syndromic surveillance [7,11,12]. However, public health professionals were not using surveillance techniques, they were informing the public about updates on specific health statuses and trends. This also allows public health professionals to take technical research and translate it into 140 characters, or lay terms, for the public to understand and disseminate among their social groups. These tweets may also contain embedded links, which then lead individuals to health department websites or other credible websites where they might not have gone to in the first place [7]. The nature of Twitter also provides the public with a way to ask questions directly, allowing public health professionals to provide information they may not have otherwise [7].

The most visible tenet of public health is the concept of social justice [18]. The concept of social justice stems directly from public health’s mission to “protect and promote health of the population as a whole” [18]. Results mirror this founding ethical principle and show that public health professionals are passionate about many health inequalities. Twitter also allows these same professionals a way to connect with other professionals, within and outside public health, who are also advocating for the same issues.

Finally, akin to public health organizations, public health professionals are also using Twitter as a means of professional development. Over the years, many states have seen significant decreases in funding for public health, severely limiting resources available to the public as well as to the public health professionals (eg, continuing education and conferences). Twitter provides public health professionals with a unique platform, to still engage with local conferences, as individuals attending tweet about sessions while they are occurring. Twitter also provides a way for public health professionals to connect and collaborate with other public health professionals in real time, despite being limited in funding for travel.

### Limitations

The scope of the tweets examined was during a 6-month window and was limited to that specific time frame. Although limited throughout the 6 months, the tweets reached saturation before 6 months, ensuring that tweets collected were representative of public health professionals’ Twitter activity. A second limitation was that the individuals analyzed only had to have a minimum of 300 followers. Although this is a small number of followers, because public health professionals are early adopters, they will not have a large following yet. The final limitation was that the category “non-public health–related” was the most common tweet category. Looking at the categories as a whole makes it appear that public health professionals are not talking about public health the majority of the time, but when one looks at the tweets (see Table 1) as two categories—non-public health and public health—11,204 tweets were on various public health topics, and only 4032 were non-public health–related.

### Conclusions

For more adoption to occur among public health professionals, public health organizations should consider removing social media filters, specifically from Twitter. The removal of social media filters would eliminate the barrier of public health professionals only being able to tweet during after work hours, thereby encouraging increased adoption of the social media platform among public health professionals as well as enabling them to rapidly spread critical health information to the public as it occurs “in real time.” Unlike the majority of health organizations, public health professionals’ individual Twitter accounts outside organizations are not monitored by the government, and they are able to disseminate important information to colleagues and the lay population, such as how climate change affects public health, that organizations may not be able to disseminate, despite how critical that information is to the overall public health in the United States.

## Abbreviations

ACA: Affordable Care Act
AIDS: acquired immunodeficiency syndrome
APHA: American Public Health Association
CDC: Centers for Disease Control and Prevention
HIV: human immunodeficiency virus
MPH: master’s in public health
NIH: National Institutes of Health
SNS: social networking site

## References

[1] Hart Mark, Stetten Nichole E, Islam Sabrina, Pizarro Katherine (2017). Twitter and Public Health (Part 1): How Individual Public Health Professionals Use Twitter for Professional Development. JMIR Public Health Surveill.

[2] (2017). Pew Internet.

[3] Perrin A (2015). Pew Internet.

[4] Java A, Song X, Finin T, Tseng B (2007). Why we twitter: understanding microblogging usage and communities. Proceedings of the 9th WebKDD and 1st SNA-KDD 2007 workshop on Web mining and social network analysis.

[5] Veltri GA (2013). Microblogging and nanotweets: Nanotechnology on Twitter. Public Underst Sci.

[6] Jansen BJ, Zhang M, Sobel K, Chowdury A (2009). Twitter power: Tweets as electronic word of mouth. J Assoc Inf Sci Tec.

[7] Bartlett C, Wurtz R (2015). Twitter and public health. J Public Health Manag Pract.

[8] (2016). Twitter.

[9] Roberts MJ, Perera M, Lawrentschuk N, Romanic D, Papa N, Bolton D (2015). Globalization of continuing professional development by journal clubs via microblogging: a systematic review. J Med Internet Res.

[10] Lefebvre C (2009). Integrating cell phones and mobile technologies into public health practice: a social marketing perspective. Health Promot Pract.

[11] Denecke K, Krieck M, Otrusina L, Smrz P, Dolog P, Nejdl W, Velasco E (2013). How to exploit twitter for public health monitoring?. Methods Inf Med.

[12] Heaivilin N, Gerbert B, Page JE, Gibbs JL (2011). Public health surveillance of dental pain via Twitter. J Dent Res.

[13] Rogers EM (2003). Diffusion of Innovations. 5th Edition.

[14] Glaser BG, Strauss AL (1967). The discovery of grounded theory: strategies for qualitative research.

[15] Patton MQ (2002). Qualitative Evaluation and Research Methods.

[16] Strauss A, Corbin JM (1990). Basics of Qualitative Research: Techniques and Procedures for Developing Grounded Theory.

[17] Marchi R (2012). With Facebook, Blogs, and Fake News, teens reject journalistic “objectivity”. J Commun Inq.

[18] Buchanan DR (2008). Autonomy, paternalism, and justice: ethical priorities in public health. Am J Public Health.

